# Age-related sensitivity deterioration evaluation of positron emission tomography utilizing cross-calibration factor measurement data

**DOI:** 10.1007/s12194-025-00882-6

**Published:** 2025-02-05

**Authors:** Asuka Kikuchi, Shoichi Watanuki, Hiroshi Watabe, Manabu Tashiro

**Affiliations:** 1 Nuclear Medicine Laboratory, Division of Short-lived Radioisotope Research, Research Center for Accelerator and Radioisotope Science, Tohoku University, 6-3 Aoba, Aramaki, Aoba-ku, Sendai, Miyagi 980-8578 Japan; 2 Radiation Protection & Safety Control Laboratory, Division of Radiation Protection and Nuclear Safety, Research Center for Accelerator and Radioisotope Science, Tohoku University, 6-3 Aoba, Aramaki, Aoba-ku, Sendai, Miyagi 980-8578 Japan

**Keywords:** Positron emission tomography (PET), Sensitivity, Cross-calibration, Long-term performance

## Abstract

Age-related deterioration in positron emission tomography (PET) systems can be monitored using cross-calibration scans for scanner calibration. This study aimed to evaluate changes in the sensitivity of a PET system over time using routinely collected cross-calibration factor (CCF) measurement data and NEMA sensitivity measurement data acquired at our facility. We used CCF measurement data acquired over eight years, from 2016 to 2023. The count rates were calculated from raw data. The NEMA sensitivity measurements were also performed in 2017 and 2024 to compare with the sensitivities obtained from the CCF measurements. The PET images were reconstructed using the CCF data. A region of interest (ROI) was placed at the center of the PET images and count rates from the PET images were obtained. The sensitivity changes in the CCF data showed a linear decrease in sensitivity over eight years, with a mean annual reduction rate of approximately 2.0%. A comparison of the NEMA sensitivity measurements indicated a decrease in sensitivity, with a 12% reduction over eight eight years. The sensitivity was higher at the center of the axial field of view than at the edges. The ROI data also showed a linear decrease in sensitivity. This is consistent with the CCF data. Additionally, the coefficient of variation increased towards the edge of the slice. By utilizing the CCF measurement data, we obtained age-related changes in the PET system, suggesting that the PET system used in our facility may experience an annual sensitivity deterioration of approximately 2.0%.

## Introduction

Positron emission tomography (PET), computed tomography (CT), and magnetic resonance imaging (MRI) have been used in clinical practice. PET provides images of biological functions, whereas CT and MRI provide anatomical images of organs [1–3]. PET is mainly used for whole-body cancer diagnosis using ^18^F-fluoro-2-deoxy-_D_-glucose [4]. Amyloid β has been found to accumulate in the brains of patients with Alzheimer’s disease, leading to the increasing use of amyloid PET for its detection [5]. Furthermore, not only amyloid β accumulation but also tau protein accumulation and neuroinflammation caused by astrocytes occur in the brains of Alzheimer's disease patients [6, 7]. The development of radiopharmaceuticals for visualizing these changes in the brain has progressed, thereby enhancing the clinical importance of PET imaging [8]. PET operates by detecting two 511 keV photons coinciding at 180°. These photons are produced by an annihilation process, in which a positron emitted by a positron-emitting radionuclide combines with an electron in the medium and is annihilated. Owing to the technical characteristics of PET systems, the imaging provides excellent quantification capabilities. The standardized uptake value (SUV) is primarily used as a semi-quantitative index in clinical practice. SUVs are used in cancer diagnosis to evaluate benign and malignant nature of tumors. By monitoring changes in the SUV over time, physicians can assess the effectiveness of treatment and detect cancer recurrence.

To ensure the quantitative accuracy of PET images, which is a key feature of PET systems, the users must perform daily quality control checks and regular cross-calibration scans. The cross-calibration scan involves determining the conversion factor that transforms PET image measurements into units of radioactivity concentration. This process enables PET images to provide quantitative data.

The performance of PET systems changes over time. Watanuki et al. [9] reported age-related changes in PET systems using data acquired from cross-calibration scans. They calculated the sensitivity and scatter fraction of the PET system from cross-calibration scans and found that the sensitivity decreased by approximately 4.7% per year, whereas the scatter fraction decreased from 4.7% to 4.1% over five years. Furthermore, this study compared performance data such as sensitivity, spatial resolution, scatter fraction, and random fraction obtained from NEMA measurements at the time of scanner installation and 10 years later. The NEMA measurements were conducted according to the NEMA NU 2-2001 protocol [10]. They reported that the NEMA sensitivity decreased by approximately 41%, and the spatial resolution of the full width at half maximum increased by an average of 1.7%. They concluded that sensitivity deterioration was more prominent in aging PET systems than in other performance parameters.

Furthermore, Matheoud et al. [11] conducted a study on age-related changes in PET/CT systems over a course of five years, reporting a gradual decline in system sensitivity. In this study, a cylindrical phantom filled with ^68^Ge radionuclide was positioned at the center of the system, and a 900 s emission scan was performed. Attenuation and scatter corrections were applied to reconstruct the data. The PET images obtained from the reconstructed data were used to calculate system sensitivity according to the NEMA NU 2-1994 protocol [12]. Based on the PET images, the results showed a 16% decrease in sensitivity over five years (average change of 3.2% per year).

Sensitivity deterioration reduces image quality, highlighting the importance of degradation trends during long-term use of PET systems. Moreover, the results from Watanuki et al. and Matheoud et al. indicated that the degree of sensitivity deterioration varied among different PET systems. Watanuki et al. have also reported that sensitivity can be determined using data from cross-calibration scans. At our facility, we collected cross-calibration scan measurement data for our PET systems (SET-3000 B/X; Shimadzu Co., Ltd., Kyoto, Japan) over a period of eight years. The scanner comprised five rings of 88 BGO (bismuth germinate) detector blocks for three-dimensional (3D) data acquisition. Each detector block has a 6 × 8 matrix of BGO crystals coupled to two photomultiplier tubes (PMTs). The total number of PMTs was 880. The BGO crystal size measured 3.5 mm (width), 6.25 mm (height), and 30 mm (depth). The detector blocks were arranged in 40 rings, each with 528 crystals, generating 79 slices. The axial field of view (FOV) of the PET scanner was 260 mm, and the transaxial FOV was 600 mm. A ^137^Cs point source was used for transmission scanning. The coincidence time window was set to 10 ns. The low- and high-energy thresholds were set to 400 and 624 keV, respectively. Prior to the clinical examination, cross-calibration factor (CCF) measurements were performed biweekly using a cylindrical phantom. The normalization measurements were conducted at the same frequency as the CCF measurements. Periodic scanner maintenance was carried out every manufacturer. The study aimed to compare the sensitivity deterioration obtained from cross-calibration scan measurements with that obtained from NEMA measurements.

## Methods

### Sensitivity

#### Cross calibration factor measurement

Cross-calibration scan data were used for retrospective evaluation of scanner sensitivity. For the CCF measurements, a cylindrical phantom with a diameter of 15 cm and a height of 30 cm was placed at the center of the scanner, which was filled with ^18^F solution of 37.0 MBq. The emission data acquisition was performed for 600 s in the 3D mode. The activity in the phantom was determined by measuring the total activity injected into the phantom using a dose calibrator. A dose calibrator was checked daily before use and a calibrated ^137^Cs coin-shaped source of 3.7 MBq was used for calibration. The dose calibrator was subjected to a linear test annually. For the linearity test, ^11^C or ^18^F were used. We followed the radioactivity decay at appropriate time intervals, ^11^C for 10 min and ^18^F for 60 min. The initial activity was set above 740 MBq and the measurement was stopped when it fell below 37 kBq. The range of the linearity test encompassed the activity levels used for CCF measurements and daily checks. The performance stability of the dose calibrator was confirmed. Random coincidence, dead time, and decay correction were applied to the raw data, and the total count of the acquired data was calculated using a scanner application. The sensitivity of the PET scanner was expressed as the count rate per unit activity concentration (cps/Bq/ml). We evaluated age-related variations in sensitivity over eight years, from 2016 to 2023. Furthermore, linear regression analysis was performed to investigate the linear decrease in sensitivity.

#### NEMA measurement

NEMA sensitivity measurements were performed to compare with the sensitivities obtained from CCF measurements. The NEMA measurements were performed according to the procedure described in NEMA NU2-2024 [13]. The line source was filled with ^18^F and sealed at both edges. The phantom was covered with five aluminum sleeves of varying thicknesses. Data were collected for a sufficient period to record at least 10,000 true coincidences per slice. PETquact (Nihon Medi-Physics Co., Ltd.) was used for data analysis [14]. As Watanuki et al. showed no significant differences between sensitivities calculated from CCF and NEMA measurements, we conducted NEMA sensitivity tests in 2017 and in 2024.

#### PET images

The PET images were reconstructed using the iterative image reconstruction method of the 3D dynamic row action maximum likelihood algorithm (3D-DRAMA) with scatter and attenuation corrections based on CCF emission data. The CCF measurements were used to determine the CCF and the PET images used in this study were not corrected for CCF. No post filters were applied. A circular region of interest (ROI) of 40 mm × 40 mm was placed at the center of each PET image. To evaluate the PET scanner sensitivity changes, the sensitivity index was calculated in cps/Bq/ml based on the ROI value in each slice. The coefficient of variation (CV) was calculated using the ROI data. We evaluated age-related variations in the sensitivity index and CV over eight years from 2016 to 2023. Furthermore, linear regression analysis was performed to investigate the linear decrease in ROI values.

## Results

### Cross calibration factor measurement

**Fig. 1:**
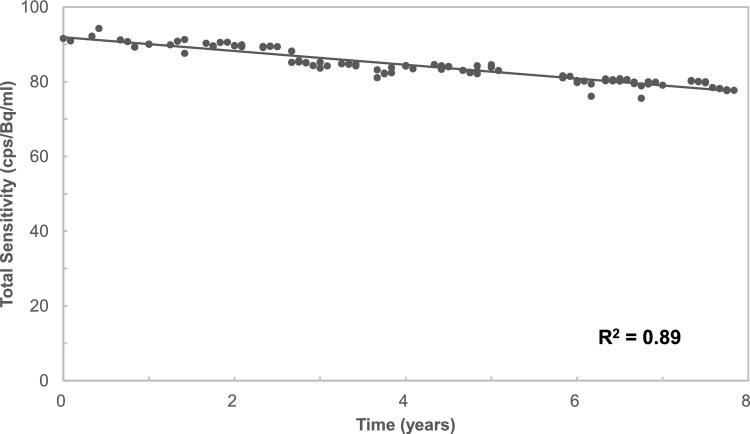
Variation in sensitivity of the PET scanner calculated from CCF measurement data over time. Sensitivity decreased gradually over eight years. Mean reduction in sensitivity was approximately 2.0% per year. The solid line was obtained by linear regression, and the coefficient of determination value was 0.89

The sensitivity changes from CCF measurements over a period of eight years (from 2016 to 2023) are shown in Fig. 1. The maximum and minimum count rates obtained were 94 and 76 cps/ml/Bq, respectively. The sensitivity decreased almost linearly over eight years and the mean reduction rate was approximately 2.0% per year. The coefficient of determination (R^2^) was 0.89. A negative correlation was observed between sensitivity and years.

### NEMA measurement

**Fig. 2:**
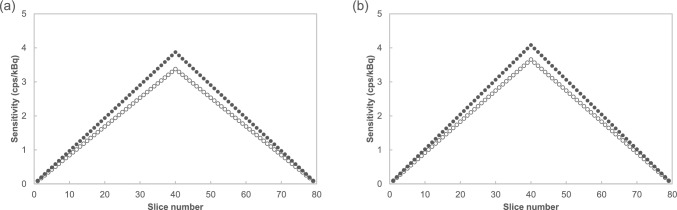
Variation in sensitivity of the PET scanner, with closed circles and open circles indicating sensitivity obtained from NEMA measurements in 2017 and 2024, respectively. The slice sensitivity in 2024 was lower than in 2017. **a** Center of the transaxial FOV, and **b** a 10 cm radial offset from the center of the transaxial FOV

The NEMA sensitivity results for 2017 and 2024 are presented in Fig. 2. Slice sensitivity increased at the center of the axial FOV compared to that at the edges of the axial FOV. Both results in the center of the transaxial FOV and a 10-cm radial offset from the center of the transaxial FOV showed that the sensitivity in 2024 decreased compared to 2017. The total sensitivity is the sum of the slice sensitivities obtained using PETquact. The average total sensitivity is the average value of the total sensitivity at the center of the transaxial FOV and a 10-cm radial offset from the center of the transaxial FOV. Table 1 summarizes the total sensitivity obtained using PETquact in 2017 and 2024. The reduction in the average total sensitivity obtained by PETquact over 8 years was 12% using NEMA sensitivity measurements. Table 2 presents the findings from various studies that evaluated age-related changes in PET scanner sensitivity.

**Table 1:** Results of NEMA sensitivity obtained by PETquact

	*S*_tot0_	*S*_tot10_	*S*_tot ave_
2017	15.5	16.3	15.9
2024	13.5	14.6	14.1
% changes	0.872	0.895	0.884

*S*_tot0_: The sum of slice sensitivity in the center of the transaxial FOV

*S*_tot10_: Sum of slice sensitivity at a 10 cm radial offset from the center of the transaxial FOV

*S*_tot ave_: The average value of *S*_tot_ and *S*_tot10_

**Table 2:** Comparisons of results of changes in sensitivity

	This study	Watanuki	Matheoud
NEMA	12%	41%	16%
	8 years	10 years	5 years
Cross calibration	2.0%	4.7%	–
	1 year	1 year	–

### PET images

**Fig. 3:**
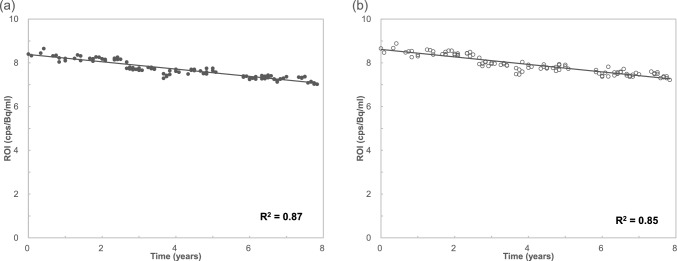
Variation in slice sensitivity index of the PET scanner calculated from ROI values of PET images over time, with closed circles and open circles indicating the sensitivity index at the center and edge of the slice, respectively. The trend in sensitivity index changes were similar. Both mean reductions in sensitivity index were approximately 2.0% per year. The solid line was obtained by linear regression, and the coefficient of determination values at the center of the slice and at the edge of 0.87 and 0.85, respectively. **a** The center of the axial FOV, and **b** the edge of the axial FOV

The changes in the sensitivity index calculated from ROI values are shown in Fig. 3. The sensitivity index decreased linearly at each slice position. The trends in the sensitivity index changed at the center and edges of the slices were similar. The mean reduction rate using the ROI data was approximately 2% per year, which was consistent with the sensitivity changes in the CCF measurement data. The R^2^ values of ROI values at center slice and edge slice were 0.87 and 0.85, respectively. A negative correlation was observed between sensitivity and years.

**Fig. 4:**
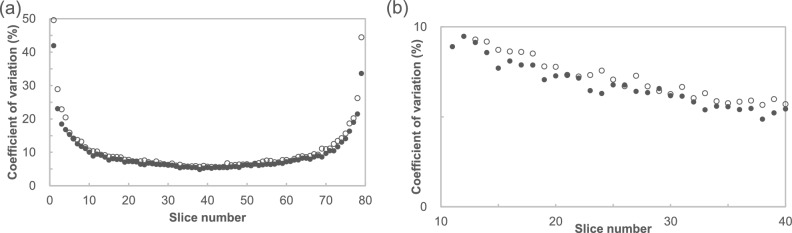
Comparison of the coefficient of variation (CV) calculated from ROI values of PET images, with closed circles and open circles indicating the CV in 2016 and 2023, respectively. The patterns of the CV changes were similar. The CV in 2023 was higher than those in 2016. **a** CV in all slice position; **b** CV from 10 to 40 slice position

The CV changes are shown in Fig. 4. The CV increased towards the edge slice, and that of the center slice was the smallest. The CV in 2023 increased compared to 2016.

## Discussion

In this study, we evaluated age-related sensitivity deterioration using data from CCF measurements, NEMA measurements, and reconstructed images. The results of the CCF measurements indicated that the sensitivity decreased by approximately 16% over eight years. NEMA sensitivity measurements were performed according to the NEMA NU2-2024 protocol for 2017 and 2024. The results from the NEMA sensitivity measurements showed that the sensitivity of the PET system used in our facility decreased by approximately 12% over 8 years, which was similar to the findings obtained from the CCF measurements. The results from Watanuki et al. also indicated that there was no difference between the sensitivity obtained from the CCF measurements and that from the NEMA measurements, validating the applicability of Watanuki et al.’s sensitivity measurement method to this study. Furthermore, the sensitivity deterioration of the PET system due to aging in our study was shown to be less pronounced compared to the results reported by Watanuki et al. and Matheoud et al. [9, 11]. Regarding the sensitivity deterioration of PET systems, Watanuki et al. reported that sensitivity decline was primarily attributed to changes in the PMT gain, specifically the decrease in the PMT gain of the detectors. This implies that the photopeak of the annihilation photons shifted out of the appropriate energy window. Uribe et al. [15] observed that the PMT gain systematically decreased by 11% over 100 days. Thus, appropriate tuning of PMTs is crucial for maintaining the sensitivity of the PET scanner. However, Zhang et al. [16] reported that the performance of their animal PET system remained stable over 6 years of use. However, this animal PET system has only 15 PMTs, which is a relatively small number compared with the human PET system. PMT adjustments in smaller systems may have contributed to this stability. Once every six months, the PET system at our facility underwent regular maintenance by the manufacturer, during which PMTs were adjusted. Our results suggest that these periodic adjustments contribute to the stability of PMT output.

A calorimeter was utilized to measure the generated heat. The calorimeters are widely applied in nuclear and particle physics to detect particles and radiation. The hadron calorimeter which is one of the calorimeters measures the energy of “hadrons,” particles made of quarks and gluons, such as protons, neutrons, pions, and kaons. The hadron calorimeters consisted of scintillators and PMTs and their components were similar to those of the PET. Age-related changes in PMTs installed in hadron calorimeters were reported by comparing the performances of new models with those of older ones [17]. The new model PMTs demonstrated stable responses and it was also observed that the responses of the new model PMTs could be clearly distinguished from those of the older models. Improvements in PMT performance can mitigate the effects of aging on the PMTs themselves. It is suggested that the sensitivity deterioration of the PET scanner at our facility which is less than that reported by Watanuki et al. may be due to the improved performance of the PMT. Kobayashi et al. [18] studied the radiation damage to a BGO scintillator. The BGO crystal was irradiated with gamma rays of ^60^Co up to a few times to investigate the output variations. Kobayashi et al. concluded that a BGO crystal has strong radiation resistance performance. Since a BGO crystal is not hygroscopic and has high stability and radiation resistance, their long-term usability is reinforced.

The results showing sensitivity changes in the PET scanner based on the count rates obtained from the PET images indicated a decline in sensitivity over time (Fig. 3). The degree of sensitivity index deterioration was approximately 2.0% per year, which was consistent with the trend observed in CCF measurement data. Owing to the characteristics of the PET system, more coincidence events were acquired closer to the center of the scanner in the 3D mode. This implies that the PET images had the least noise in the central slices, with the noise increasing towards the edges. Consequently, the CV values of the central slices were lower than those of the edge slices (Fig. 4a). In other words, the ROI values in the slices farther from the center showed greater variability than those in the central slices. By contrast, the trends in the count rate changes calculated from the central and edge slices were similar. Although the count rates calculated from images at the same slice position could be used to assess the aging of the PET system, the variability in the count rates increased at the edges of the FOV (Fig. 4b). Therefore, evaluating sensitivity changes in the central FOV is recommended for more accurate assessments.

PET image reconstruction incorporates several factors such as CCF and normalization scans. Normalization is the standard technique for correcting detector efficiency variations and geometrical effects in each detector pair. As the sensitivity response profile is not constant in the axial direction, where it peaks at the center of the FOV, a method must be adopted to remove this effect in the reconstruction procedure. The CCF corrected the sensitivity variation along the axial FOV. By incorporating normalization and CCF into the reconstruction, the pixel values in the PET image were corrected for sensitivity variations and converted into absolute activity units. CCF measurements were used to determine the CCF, and the PET images used in this study were not corrected for CCF. We believe that PET images without CCF reflect changes in sensitivity. The error components of these factors can influence the count rates calculated from the ROI, resulting in count rates derived from PET images containing many error components. Consequently, the count rates from PET images are likely to be more variable than those obtained from CCF or NEMA measurements. Therefore, when evaluating sensitivity changes using cross-calibration scan data, it is preferable to use count rates obtained from the raw data rather than those from the reconstructed images. Commercial PET scanners equipped with silicon photomultiplier (SiPM) detectors have also been developed. The properties of the SiPMs differ from those of conventional PMT. It is anticipated that the trend of sensitivity deterioration in SiPM-based PET scanners owing to aging is inconsistent with our results. Further studies on long term performance of age-related changes in PET systems are required.

One limitation of this study is that sensitivity evaluation using reconstructed images employed an iterative reconstruction method. This method is commonly used for routine examinations at our facility. Although various PET image reconstruction methods are available, different reconstruction algorithms can result in varying pixel values, even for the same raw data. Understanding the influence of reconstruction methods on sensitivity changes over time represents an important area for future research.

## Conclusion

This study investigated long-term sensitivity changes in a PET system using CCF measurements and reconstructed images. The trend of age-related changes in sensitivity was captured using the CCF measurements and evaluated based on the count rate calculated from the reconstructed images. The sensitivity deterioration results obtained from the CCF and PET images were similar to those obtained from NEMA measurements. Our findings the use of CCF measurements for assessing long-term sensitivity changes, demonstrating that the PET scanner at our facility showed an annual decrease in sensitivity of approximately 2.0%.

## Data Availability

The data used to support the findings of this study are available from the corresponding author upon reasonable request.
